# Neuroprotective effect of ketamine against TNF‐α‐induced necroptosis in hippocampal neurons

**DOI:** 10.1111/jcmm.16426

**Published:** 2021-03-03

**Authors:** Lu Wang, Bin Deng, Panpan Yan, Huanghui Wu, Chunhui Li, Hongrui Zhu, Jiwei Du, Lichao Hou

**Affiliations:** ^1^ Department of Anesthesiology Xiang'an Hospital of Xiamen University School of Medicine Xiamen University Xiamen China; ^2^ State Key Laboratory of Cellular Stress Biology Xiamen University Xiamen China; ^3^ Medical College of Yan'an University Yan'an China; ^4^ Department of Nursing Xiang'an Hospital of Xiamen University School of Medicine Xiamen University Xiamen China

**Keywords:** ketamine, necroptosis, reactive oxygen species, systemic inflammatory response syndrome, tumour necrosis factor‐α

## Abstract

Tumour necrosis factor‐α (TNF‐α), a crucial cytokine, has various homeostatic and pathogenic bioactivities. The aim of this study was to assess the neuroprotective effect of ketamine against TNF‐α‐induced motor dysfunction and neuronal necroptosis in male C57BL/6J mice in vivo and HT‐22 cell lines in vitro. The behavioural testing results of the present study indicate that ketamine ameliorated TNF‐α‐induced neurological dysfunction. Moreover, immunohistochemical staining results showed that TNF‐α‐induced brain dysfunction was caused by necroptosis and microglial activation, which could be attenuated by ketamine pre‐treatment inhibiting reactive oxygen species production and mixed lineage kinase domain‐like phosphorylation in hippocampal neurons. Therefore, we concluded that ketamine may have neuroprotective effects as a potent inhibitor of necroptosis, which provides a new theoretical and experimental basis for the application of ketamine in TNF‐α‐induced necroptosis‐associated diseases.

## 1 INTRODUCTION

Systemic inflammatory response syndrome (SIRS) is caused by the activation of the innate immune system and results in the stimulation of excessive inflammatory responses, and the production and secretion of pro‐inflammatory cytokines, such as tumour necrosis factor‐α (TNF‐α), and reactive oxygen intermediates.^1^, ^2^ In addition, the disbalanced and dysregulated inflammatory response also causes sepsis, which may lead to life‐threatening organ dysfunction.^3^ Statistically, sepsis developing from SIRS affects ~18 million people worldwide; it is a common cause of acute and severe diseases, and sepsis‐associated mortality rates range from 35% to 55%. Survivors often have a poor prognosis, which seriously affects the quality of life.^4^ Evidence from previous studies indicates that SIRS can lead to cognitive impairment, physical disability or even sepsis‐associated encephalopathy^5^; however, the underlying pathogenic mechanisms remain to be fully elucidated. TNF‐α is as a pleiotropic factor that plays both homeostatic and pathophysiological roles in the central nervous system (CNS),^6^ and is mainly generated by activated microglia and astrocytes in response to various stimuli related to infection or injury. For example, it has been reported that necroptosis could be activated in the mouse hippocampus by intracerebroventricular injection of TNF‐α.^7^ The TNF receptor family‐mediated necroptosis signalling pathway requires the activation of receptor‐interacting protein kinase 1 (RIPK1), which subsequently recruits and activates the kinase receptor interacting serine/threonine kinase 3 (RIPK3).^8^, ^9^ Activated RIPK3 phosphorylates its substrate, mixed lineage kinase domain‐like protein (MLKL), which can oligomerize and translocate from the cytosol to the membranes to lead to membrane disintegration, resulting in necrosis.^10^, ^11^ The aim of this study was to examine whether necroptosis of hippocampal neurons is induced in an in vivo experimental model of SIRS,^12^, ^13^, ^14^ created by injecting TNF‐α intravenously.

As a non‐competitive glutamatergic N‐methyl‐D‐aspartate receptor antagonist, ketamine has been extensively used as a clinical anaesthetic and analgesic.^15^ Recent studies have also demonstrated that ketamine exerts rapid antidepressant,^16^ anti‐inflammatory and immunomodulatory effects.^17^, ^18^ In addition, evidence shows that ketamine may also reverse synaptic deficits and induce synaptogenesis, exhibiting neuroprotective effects.^19^

In the present study, the effects of ketamine on TNF‐α‐induced necroptosis were examined using cultured cells and a mouse model of TNF‐α‐induced SIRS. We also evaluated the effects of ketamine on the long‐term physical functions of post‐TNF‐α mice, hippocampal damage, neuronal loss, and oxidative stress using the open field test, Nissl staining, immunofluorescence, flow cytometry and western blotting. Furthermore, the present study addressed the possible contribution of the clinical transformation of ketamine to the pathological mechanism in the SIRS model.

## 2 MATERIALS AND METHODS

### 2.1 Animals

Adult male C57BL/6J wild‐type mice (18‐25 g; Shanghai SLAC Laboratory Animal Co, Ltd.) were bred in specific pathogen‐free conditions, and housed in air‐conditioned, temperature‐controlled rooms with a 12 hours/12h light/dark cycle (lights on, 08:00 am), 22‐25°C ambient temperature, and ad libitum access to food and water in the Laboratory Animal Center of Xiamen University. Prior to experimentation, animals were allowed to habituate to the new housing environment for 7 days. All procedures and animal use were approved by the Animal Ethics Committee of Xiamen University (Approval No. XMULAC20190054). Every effort was made to minimize stress to the animals.

### 2.2 Animal grouping and experimental protocol

**FIGURE 1:**
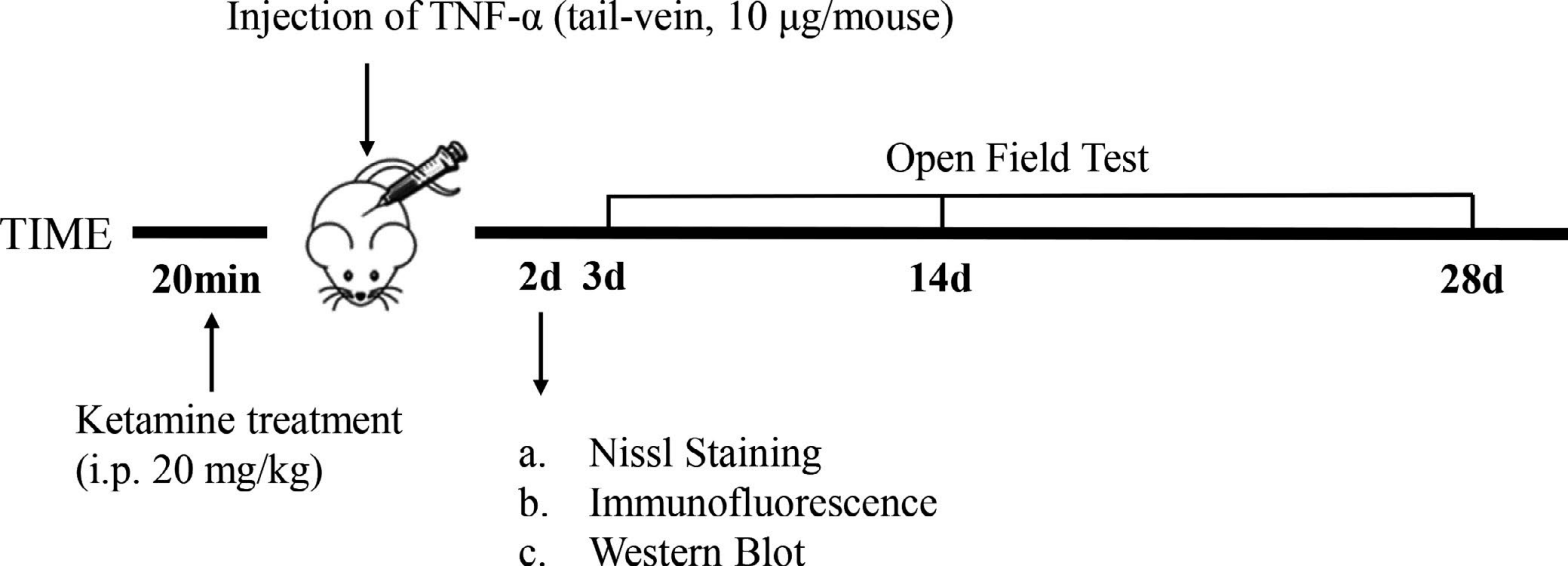
A flow diagram showing the experimental design. The SIRS model was induced on male C57BL/6J mice intravenously received murine TNF‐α (10 μg/mouse). Mice received an injection of 20 mg/kg ketamine 20 min before the administration of TNF‐α. On the 3th, 14th and 28th day after TNF‐α induction, subgroups of mice treated with ketamine started performing the open field test along with TNF‐α mice and normal mice. On the 2th day, some mice were euthanized via intracardiac perfusion for Nissl staining and immunohistochemical staining; the rest of the mice were euthanized for biochemical and molecular biological studies

The mice were randomly assigned into three groups treated as follows—TNF‐α group: mice intravenously received murine TNF‐α (eBioscience) (10 μg/mouse) in 200 μL of endotoxin‐free phosphate‐buffered saline (PBS) (pH 6.8); ketamine pre‐treated group: mice were intraperitoneally injected with ketamine (cat. no. 1707031; Gutian Pharmaceutical Co. Ltd.) (20 mg/kg) 20 minutes prior to TNF‐α injection; and control group: mice intravenously received an equal volume of Dulbecco's PBS (vehicle) 20 minutes before the challenge with TNF‐α (Figure 1).

### 2.3 Open field test

The open field apparatus consisted of a square arena (50 × 50 cm) and 50 cm high walls made of grey polyvinyl chloride plastic. On the day of the test, mice were transported to the testing room and left in their home cages for 1 hour prior to testing. At the initiation of each session, a mouse was placed in a particular corner of the arena and allowed to explore for 5 minutes. The apparatus was cleaned with 70% ethanol prior to testing each animal. Time was subsequently recorded, and each mouse was allowed to explore the testing area for 10 minutes. The statistical data were recorded and analysed using the Noldus EthoVision XT system (Ugo Basile SLE).

### 2.4 Nissl staining

The mice were sacrificed under isoflurane anaesthesia 48 hours after tail‐vein injection of TNF‐α, and then perfused through the ascending aorta with 100 mL of normal saline followed by 100 mL of 4% (w/v) paraformaldehyde in 0.1 M PBS (pH 7.4). The brain of each mouse was dissected and removed from the skull, and resected‐brain tissue was fixed in 4% paraformaldehyde for 24 hours at 4°C. Tissues were subsequently stored overnight in 30% sucrose phosphate buffer until the tissue sank to the bottom of the solution. Tissue sections (8 μm) were cut in the coronal plane using a freezing microtome (CM19500; Leica Microsystems, Inc) and mounted on gelatine‐coated slides. The sections were then stained with 0.1% cresyl violet solution (Sigma‐Aldrich; Merck KGaA) at 37°C for 30 minutes. The sections were subsequently rinsed in distilled water, rehydrated using a descending alcohol series, and checked microscopically for optimal results. Tissues were dehydrated in 100% ethanol, washed in xylene and finally scanned using an Olympus BX53 Scanner (Olympus Corporation) at a magnification of ×20/×4.

### 2.5 Immunofluorescence

Brain tissue containing the hippocampus was embedded in optimal cutting temperature compound and then cut into thick coronal sections (30 μm) using a freezing microtome. Sections were collected in dishes containing 0.01 M PBS. Free‐floating sections were permeabilized in permeabilization buffer containing 0.25% (v/v) Triton X‐100 (Beyotime Institute of Biotechnology) for 15 minutes and subsequently blocked for 30 minutes with 3% bovine serum albumin (Beyotime Institute of Biotechnology) in 0.01 M PBS with gentle agitation. Tissues were subsequently incubated with the following primary antibodies: mouse anti‐RNA binding Fox‐1 homolog 3 (NeuN) (dilution, 1:1,000; cat. no., ab104224; Abcam), rabbit anti‐MLKL (phospho S345) (dilution, 1:400; cat. no., ab196436; Abcam), rabbit anti‐ionized calcium binding adaptor molecule 1 (Iba1) (dilution, 1:400; cat. no., ab178847; Abcam) and rat anti‐CD68 (dilution, 1:500; cat. no., ab53444; Abcam) in primary antibody dilution medium (Beyotime Institute of Biotechnology) overnight at 4°C. The sections were then incubated with a mixture of the following secondary antibodies: Alexa 488‐donkey anti‐mouse IgG (cat. no., ab150061; Abcam); Alexa 594‐donkey anti‐rabbit IgG (cat. no., ab150064; Abcam); Alexa 488‐donkey anti‐rabbit IgG (cat. no., ab150061; Abcam), and Alexa 594‐donkey anti‐rat IgG (cat. no., ab150156; Abcam) in secondary antibody dilution medium (Beyotime Institute of Biotechnology) for 2 hours at room temperature. Thereafter, sections were mounted onto gelatine‐coated glass slides, air‐dried and cover‐slipped with anti‐fade mounting medium with dihydrochloride (Beyotime Institute of Biotechnology). Samples were gently washed thrice with 0.01 M PBS for 10 minutes before each step. Sections were observed using a fluorescence microscope (cat. no., FV1000MPE‐B; Olympus Corporation).

### 2.6 Cell culture and reagents

Mouse hippocampal HT‐22 cells were cultured in DMEM (cat. no., 11995065, Gibco; Thermo Fisher Scientific, Inc) supplemented with 10% foetal bovine serum (cat. no., 10091148 Gibco; Thermo Fisher Scientific, Inc) and 1% antibiotic/antimycotic (cat. no., 15140122 Gibco; Thermo Fisher Scientific, Inc) at 37°C in 5% CO_2_. Recombinant TNF‐α was purified as described previously.^8^ z‐VAD was purchased from Merck Millipore, and propidium iodide was purchased from Sigma‐Aldrich (Merck KGaA). The following antibodies were used for western blotting: anti‐RIP1 (cat. no. 3493s; Cell Signaling Technologies, Inc), anti‐p‐MLKL (cat. no., ab196436; Abcam) and anti‐GAPDH (cat. no., AC033; AbClon, Inc).

### 2.7 Cell viability assay

HT‐22 cells were treated with the indicated drug for ~12 hours. Cell survival was determined using the Cell Titer‐Glo Luminescent Cell Viability Assay kit (Promega Corporation) according to the manufacturer's protocols. Luminescence was recorded using the Omega POLAR Star (BMG Labtech GmbH).

### 2.8 Reactive oxygen species (ROS) detection

Reactive oxygen species levels were measured using a fluorometric intracellular ROS kit (cat. no., MAK143; Sigma‐Aldrich; Merck KGaA), according to the manufacturer's protocols. Stained cells were viewed using a fluorescence microscope. Cells were treated with TNF‐α/z‐VAD or TNF‐α/z‐VAD/ketamine for 3 hours, and fluorescence was measured using a CytoFLEXS flow cytometer (Beckman Coulter, Inc).

### 2.9 Western blot analysis

HT‐22 cells were collected and centrifuged in 0.01 M PBS solution, and the supernatant was removed. The pellets were dissolved in PRO‐PREP solution by vortexing and ultrasonication for western blot analysis. Briefly, proteins were separated using 10% SDS‐PAGE gels along with the marker (26616; Thermo Fisher Scientific, Inc) and then transferred onto PVDF membranes (cat. no., ab133411; Abcam). The membranes were blocked using 5% skim milk for 40 minutes to reduce non‐specific binding and then incubated with primary antibodies (dilution, 1:1,000) at 4°C overnight. Subsequent to being incubated with mice (cat. no., S0100; Beijing Lablead Biotech Co., Ltd.)/rabbit (cat. no., S0101; Beijing Lablead Biotech Co., Ltd.) horseradish peroxidase–conjugated secondary antibodies (dilution, 1:1,000) for 2 hours at room temperature, protein bands were visualized using an enhanced chemiluminescence detection kit (cat. no., E1060; Beijing Lablead Biotech Co., Ltd.). ImageJ software (National Institutes of Health) was used to determine the band intensities to reflect the expression of each protein.

### 2.10 Statistical analysis

Independent experiments were performed in duplicate or triplicate, and data were analysed using GraphPad Prism 7.0 (GraphPad Software, Inc). Data are presented as mean ± standard error of the mean unless indicated otherwise. One‐way analysis of variance and the Bonferroni multiple comparison post hoc test were used. *P* <.05 was considered to indicate a statistically significant difference.

## 3 RESULTS

### 3.1 Result 1. TNF‐α‐induced motor dysfunction is attenuated by pre‐treatment with ketamine

**FIGURE 2:**
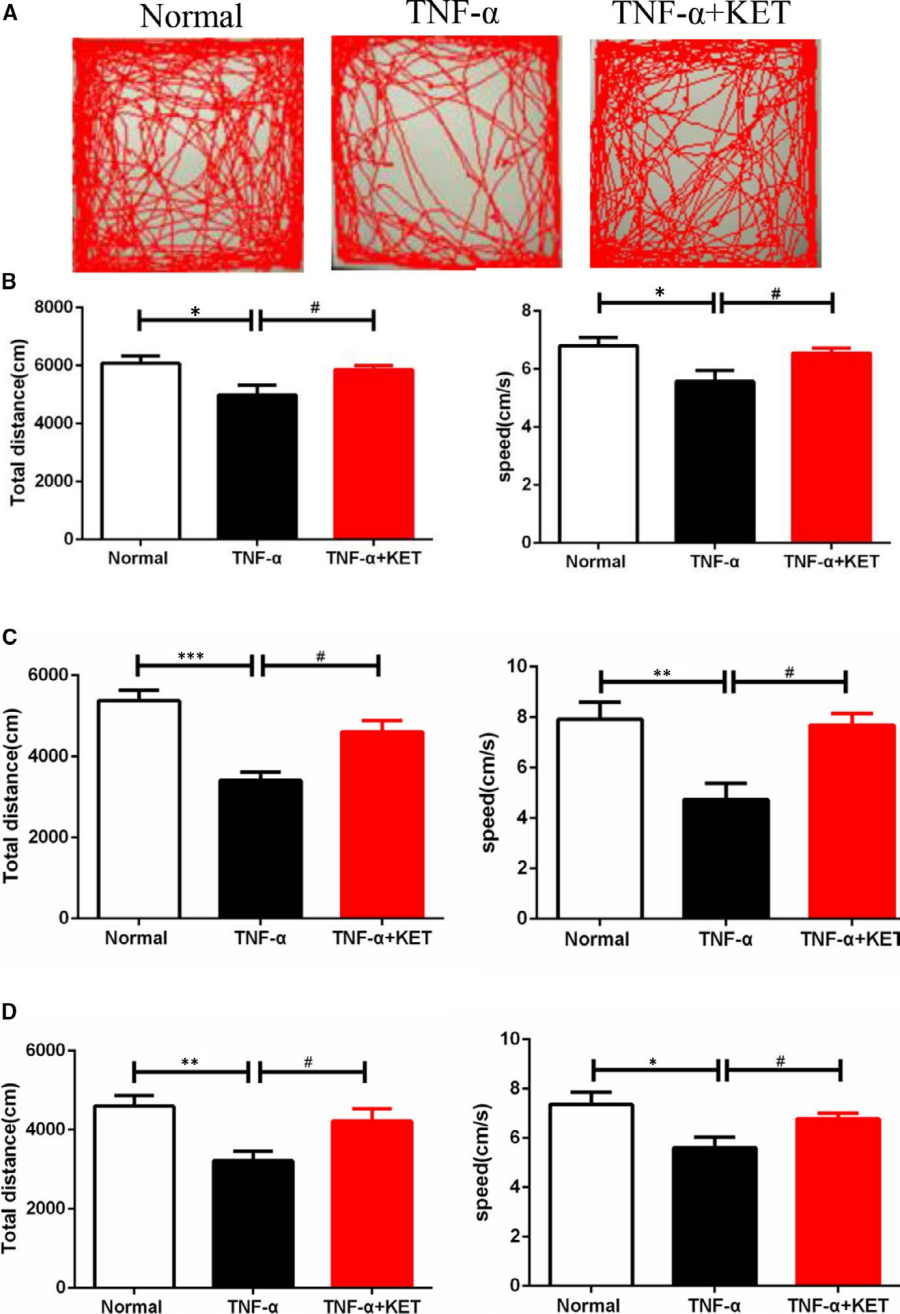
Ketamine alleviated motor dysfunction caused by TNF α‐induced SIRS. (A) The trajectory, (B‐D) the total motion distance and the average motion speed in the open field test after 3, 14 and 28 d following tale‐vein TNF‐ α injection and pre‐treatment with ketamine. All data were shown as mean ± s.e.m. n = 7/group, * significantly different from the normal group; # significantly different from the TNF ‐α group. *P <.05 and #*P* <.05

Tumour necrosis factor‐α is a crucial mediator of neuroinflammation, and elevated levels of TNF‐α are associated with various neurodegenerative conditions and contribute to neurotoxicity.^20^ As TNF‐α‐induced SIRS represents an acute model, mimicking a cytokine storm and inducing tissue damage only a few hours after TNF‐α injection, tail‐vein injection with a non‐lethal dose of TNF‐α was performed (Figure S1), to observe the behaviour of the surviving mice. The effect of TNF‐α on the activity levels was examined using the open field test. Analysis of the trajectories of the mice (Figure 2A) revealed that systemic TNF‐α administration reduced the total distance travelled as well as the speed of movement of mice, which were attenuated by ketamine pre‐treatment within 3 days (Figure 2B), 14 days (Figure 2C) and 28 days (Figure 2D). In addition, systemic administration of TNF‐α‐induced a long‐term deficit in activity levels, while there were no deficits in similarly challenged ketamine pre‐treatment animals (Figure 2).

### 3.2 Result 2. effect of ketamine on TNF‐α‐induced necroptosis in hippocampal neurons in vivo

**FIGURE 3:**
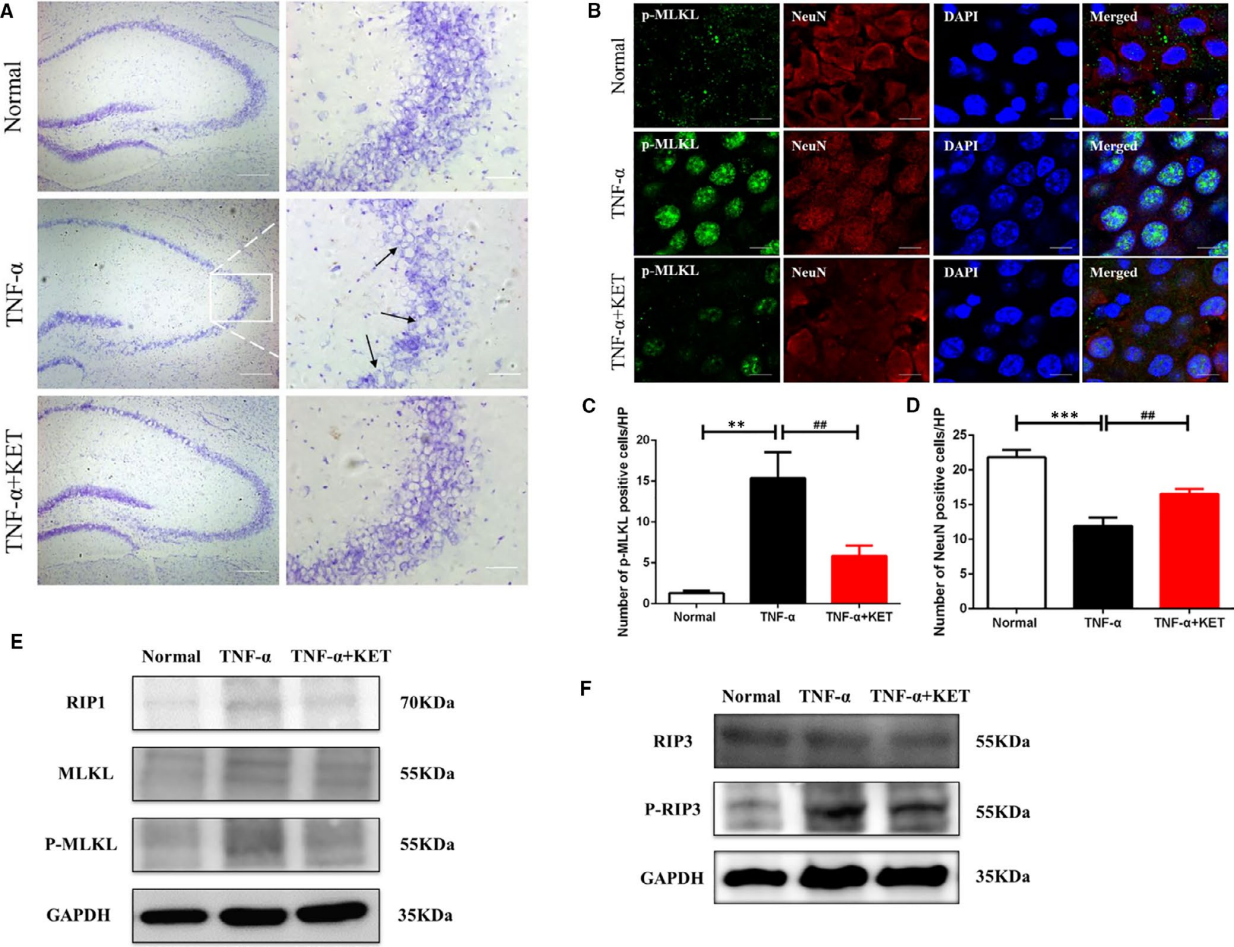
Ketamine attenuated TNF‐α‐induced neuronal necroptosis in vivo. (A) Representative images of the Nissl staining in the Carbonic Anhydrase 3 region of the hippocampus. (B‐C) Representative confocal images and the quantified histogram of p‐MLKL and NeuN positive cells in the hippocampus. (D) Western blot analysis of necroptosis markers RIP1, MLKL, RIP3, p‐MLKL and p‐RIP3 in the hippocampus. Magnification, ×80. Scale bars = 200 µm (Left) and 50 µm (Right) in A, Scale bars = 40 µm in B. All data were shown as mean ±  s.e.m. n  = 7/group, * significantly different from the normal group; # significantly different from the TNF ‐α group. *P <.05, ##*P* <.01 and ###*P* <.001

To further explore whether TNF‐α‐induced motor dysfunction was associated with brain injury, Nissl staining of neurons was performed to evaluate neurological dysfunction and TNF‐α‐induced neuronal damage. It was observed that administration of TNF‐α caused a reduction in neuronal density in the hippocampus, particularly in the carbonic anhydrase 3 region. At the same time, pre‐treatment with ketamine prevented the TNF‐α‐induced loss of hippocampal neuron number and density in vivo (Figure 3A). We also demonstrated that this loss was caused by necroptosis, and immunofluorescence revealed that ketamine pre‐treatment could significantly increase the number of NeuN‐positive neurons in the hippocampal region (Figure 3B,C,D). Meanwhile, p‐MLKL and p‐RIP3 levels were markedly decreased in the hippocampus after ketamine pre‐treatment (Figure 3E,F). These results clearly indicate that ketamine may have a novel function in SIRS as a potent inhibitor of necroptosis.

### 3.3 Result 3. TNF‐α‐induced microglial activation in the hippocampus is attenuated by ketamine pre‐treatment

**FIGURE 4:**
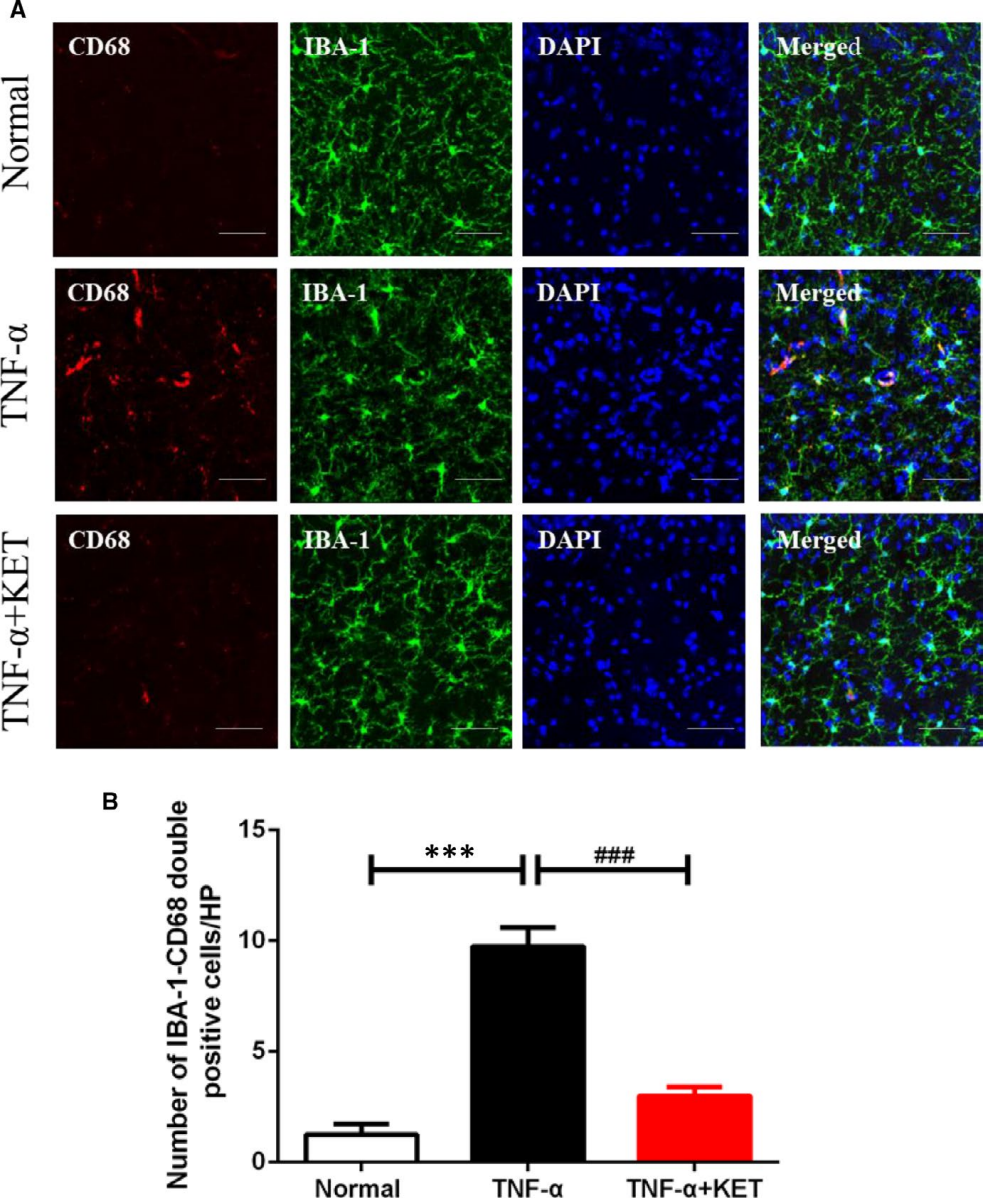
Ketamine suppressed TNF‐α‐induced microglia activation in hippocampus. (A) Confocal images and (B) immunofluorescence analysis data showing Iba‐1 and CD68 double‐labelled positive cells in the hippocampus. Scale bars = 40 µm. All data were shown as mean ± s.e.m. n = 7/group, *significantly different from the normal group; # significantly different from the TNF ‐α group. ***P <.001 and ###*P* <.001

The effect of a single systemic challenge with TNF‐α on C57BL/6J mice was examined using the microglial activation markers Iba‐1 and CD68. Confocal microscopy results showed an increase in Iba‐1‐CD68 double‐positive cells after TNF‐α administration, which was attenuated by ketamine pre‐treatment (Figure 4A,B). Overall, ketamine may inhibit the activation of microglia in TNF‐α‐treated mice, which would contribute to its anti‐inflammatory potential.

### 3.4 Result 4. Ketamine improved the survival of HT‐22 hippocampal neuronal cells

**FIGURE 5:**
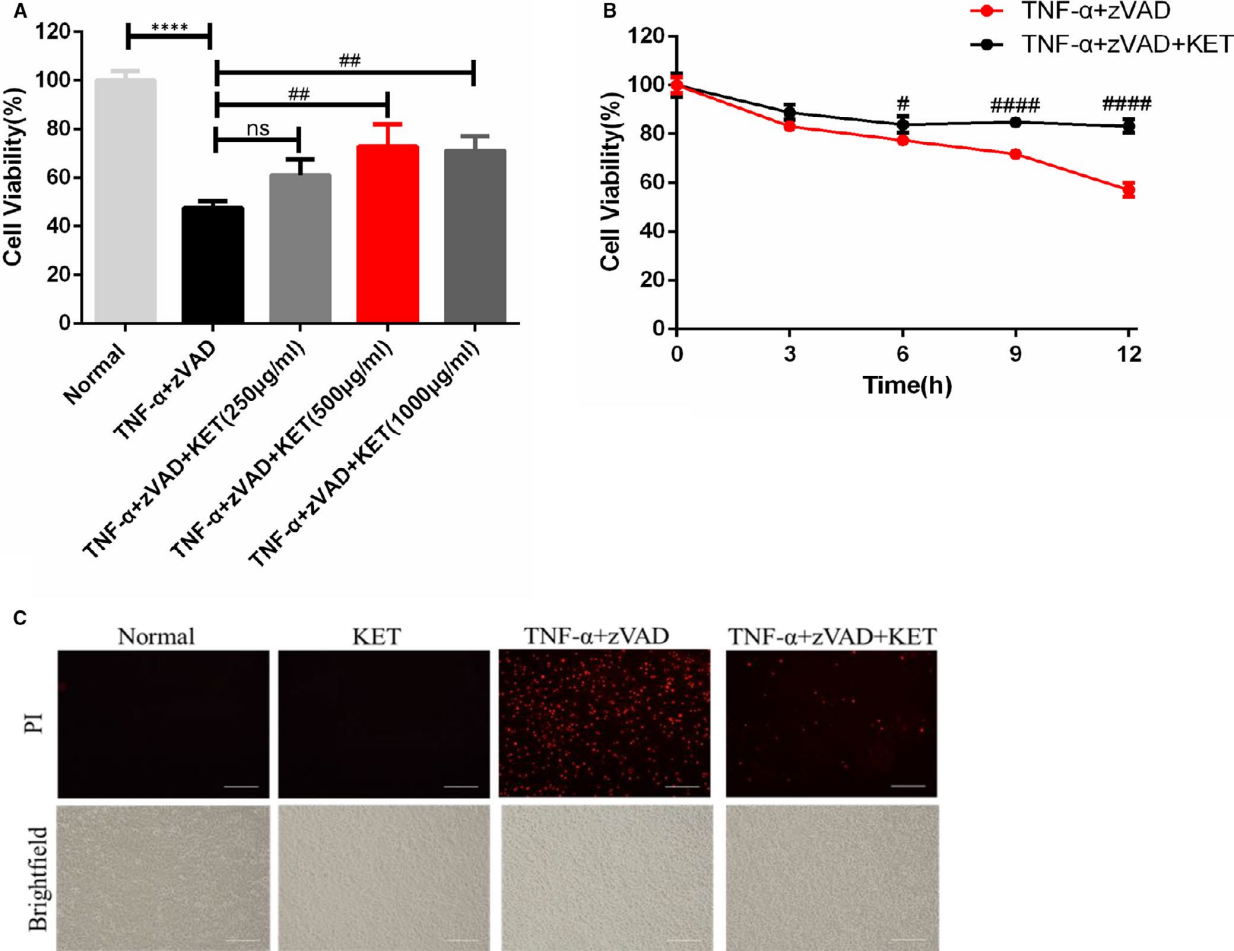
Ketamine improved the survival of HT‐22 hippocampal neuron cells. (A‐B) Cell viability via measuring ATP level in HT‐22 cells. (C) Immunofluorescence PI were detected in HT‐22 cells. Scale bars = 100 µm. All data were shown as mean ± s.e.m. *Significantly different from the normal group; #significantly different from the TNF‐α/z‐VAD group. ****P <.0001, ##*P* <.01 and ####*P* <.0001. All experiments were repeated three times with similar results

The subsequent experiments were designed to elucidate the modulatory effects of ketamine on the necroptosis signalling cascade. HT‐22 hippocampal cells have been reported to be sensitive to TNF‐α only upon caspase blockage and subsequently undergo necroptosis.^7^ In this study, we first evaluated the effects of ketamine against TNF‐α (10 ng/mL)/z‐VAD (20 μmol/L) administration on cell viability by measuring ATP levels in HT‐22 cells. The results showed that TNF‐α/z‐VAD administration significantly reduced cell viability, and ketamine markedly reduced TNF‐α/z‐VAD‐induced cell toxicity in HT‐22 cells in a dose‐ and time‐dependent manner (Figure 5A,B). Thereafter, 500 μg/mL of ketamine was selected as a major dose to explore its effects on TNF‐α‐induced neurotoxicity in HT‐22 cells in vitro. After 4 hours, analysis of propidium iodide‐positive HT‐22 cells treated with TNF‐α/z‐VAD/ketamine (500 μg/mL) indicated that ketamine prevented these cells from losing membrane permeability and undergoing necroptosis (Figure 5C).

### 3.5 Result 5. Treatment with ketamine exerted neuroprotective effects by inhibiting ROS accumulation and suppressing TNF‐α‐induced necroptosis of HT‐22 hippocampal neuronal cells

**FIGURE 6:**
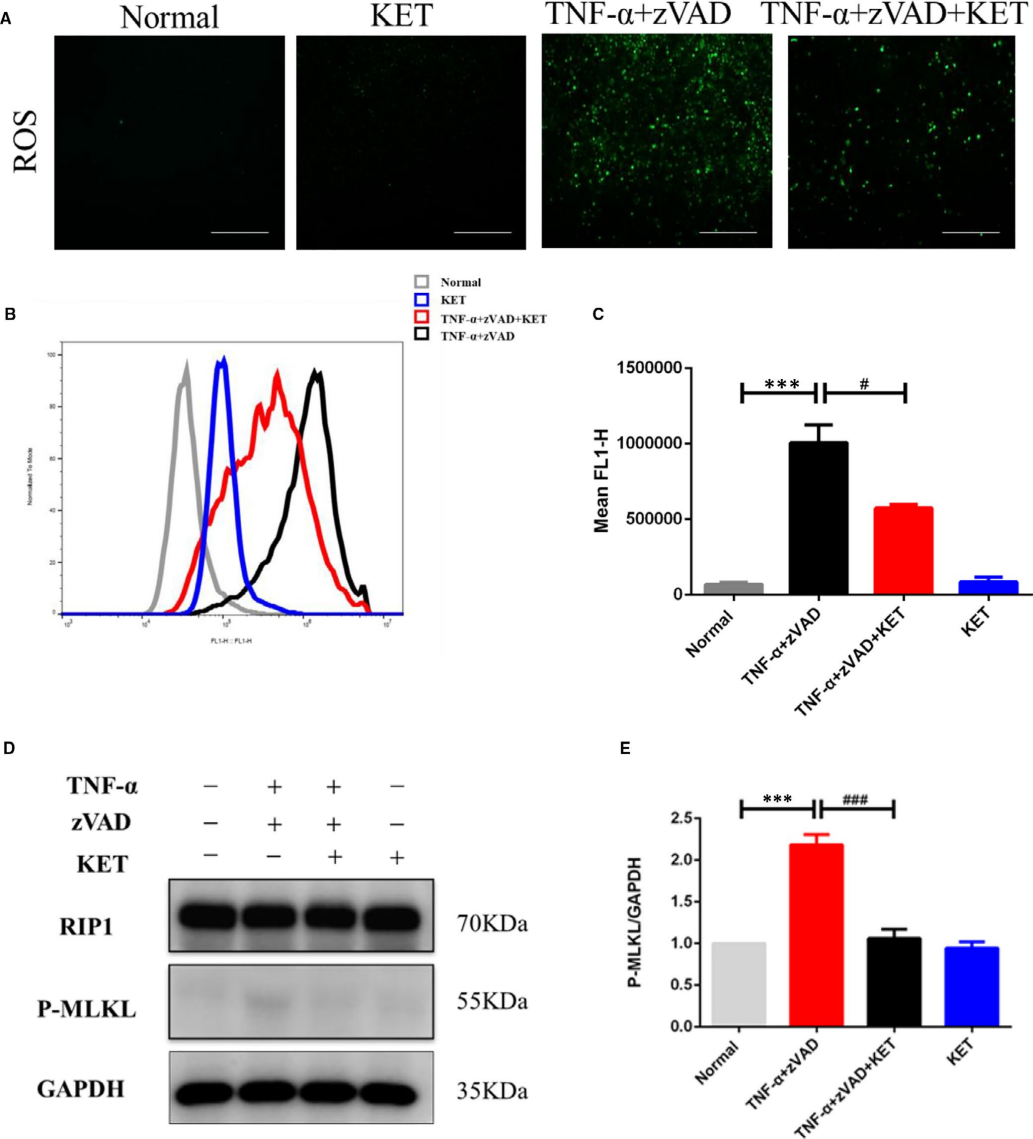
Ketamine suppressed TNF‐α ‐induced necroptosis of HT‐22 hippocampal neuron cells. (A‐C) Representative images of production of ROS and quantitative analysis by flow cytometry. (D‐E) Western blot analysis of necroptosis markers RIP1 and p‐MLKL in HT‐22 cells. Scale bars = 100 µm. All data were shown as mean ± s.e.m. *Significantly different from the normal group; #significantly different from the TNF‐α/z‐VAD group. ***P <.001, #*P* <.05 and ###*P* <.001. All experiments were repeated three times with similar results

In vitro ROS assays revealed that after 4 hours, there was enhanced expression of ROS in TNF‐α/z‐VAD‐treated HT‐22 cells compared with that in the control group (Figure 6A). In addition, flow cytometry results indicated that ROS levels were significantly downregulated in the TNF‐α/z‐VAD/ketamine‐treated cells (Figure 6B,C). Finally, to assess the effects of TNF‐α on the expression of RIP1 and p‐MLKL, western blotting analysis was performed, which revealed that p‐MLKL expression was significantly upregulated in TNF‐α/z‐VAD‐treated cells, and significantly downregulated in TNF‐α/z‐VAD/ketamine‐treated cells (Figure 6D,E). Overall, these results support the hypothesis that ketamine suppressed TNF‐α‐induced necroptosis of HT‐22 hippocampal neuronal cells by inhibiting ROS accumulation and MLKL phosphorylation.

## 4 DISCUSSION

The present study revealed that surviving mice with TNF‐α‐induced SIRS had motor function decline, and the open field test data indicated that these mice had problems related to anxiety and exploration. However, based solely on the aforementioned observations, it could not be discerned whether this phenomenon was a result of decreased activity levels caused by dyskinesia. Furthermore, TNF‐α‐induced motor dysfunction was attenuated by pre‐treatment with ketamine. We also demonstrated that TNF‐α‐induced brain injury led to neuronal necroptosis and microglial activation in C57BL/6J mice. Collectively, these data indicate that the neuroprotective effect of ketamine against TNF‐α‐induced necroptosis was mediated via inhibition of ROS production and MLKL phosphorylation in hippocampal neurons.

Systemic inflammatory response syndrome, an over‐reactive immuno‐inflammatory response, represents a significant disease burden and is associated with long‐term physical, cognitive and psychosocial morbidity.^21^ Previous studies suggested that a high intravenous dose of TNF‐α (>10 μg) promoted mouse death within 24‐36 hours,^22^, ^23^ while our data suggested that a sub‐lethal dose (5‐10 μg) dramatically induced long‐lasting sterile pathological SIRS‐like effects, with motion‐related dysfunction and susceptible tissue damage, even in the brain. With the impaired structure and function of the blood‐brain barrier induced by intravenous TNF‐α administration,^24^ peripheral immuno‐inflammatory dysfunction would ignite intense central neuroinflammation in a TNF‐α‐dependent and/or independent manner. Necroptosis, or caspase‐independent programmed cell death, is known to be involved in various pathological conditions, including TNF‐α‐induced peripheral and central inflammatory processes, both in vivo and in vitro.^25^ Recent studies identified that the activation of RIPK1, RIPK3 and MLKL is involved in necroptosis,^8^, ^26^ and provided evidence of the signalling events of TNF‐α‐initiated neurotoxicity being mediated by RIPK1‐RIPK3‐MLKL both in the mouse hippocampus after intracerebroventricular injection of TNF‐α and in HT‐22 hippocampal neuronal cells with TNF‐α incubation.^7^ Considering that some studies have reported that RIPK3 has a pro‐inflammatory effect independent of its role in necroptosis, the pseudokinase MLKL is currently regarded as the sole and prime effector of necroptosis, which terminates in the rupture of the plasma membrane and the leakage of intracellular contents from apoptotic cells. During necroptosis, MLKL is a functional substrate of RIPK3. Upon phosphorylation by RIPK3, MLKL forms oligomers and translocates to the plasma membrane.^27^ Our data from the present study strongly support the notion that the TNF‐α‐initiated toxic effects on hippocampal neurons and their subsequent loss was caused by necroptosis, as indicated by RIPK1‐RIPK3‐MLKL phosphorylation and signalling events after tail vein injection of a sub‐lethal dose of TNF‐α.

Classically, necroptotic cell death is known to be characterized by disrupted plasma membrane; however, the downstream events leading to membrane collapse are far from being clarified. ROS production and accumulation have been suggested to be required for necroptosis in cells such as L929, the human 5‐8F NPC cell line and HT‐29 human colon cancer cells.^28^, ^29^, ^30^ In the present study, we observed increased ROS production and accumulation in TNF‐α‐induced necroptotic HT‐22 mouse hippocampal neurons; however, further studies are required to identify the relationship between ROS and TNF‐α‐induced necroptosis as well as to clarify crucial downstream events required for causing this necroptosis of hippocampal neurons.

Ketamine is a traditional narcotic analgesic and psychotomimetic drug with abuse potential in medical practice. It was considered a notable and attractive “drug of the year” when a rapid and sustained anti‐depressant profile, with selective rescue of eliminated spines and restoration of coordinated activity in multicellular ensembles, was revealed.^31^, ^32^ However, a growing body of evidence indicates that a sub‐anaesthetic dose of ketamine (5‐30 mg/kg) exerts immuno‐inflammatory modulation in sepsis, ischaemia‐reperfusion, and a burn injury rodent models.^33^, ^34^ Interestingly, our data challenge the generally held view of the role of ketamine as an anti‐inflammatory agent when used both clinically^35^, ^36^, ^37^ and experimentally.^38^, ^39^, ^40^ Our data showed that ketamine alleviated TNF‐α‐induced motor dysfunction in a dose‐dependent manner that a dose of 20 mg/kg could significantly improve the activity and speed of mice (Figure S2). Meanwhile, we also demonstrated that the protection provided by ketamine was by inhibiting the expression of p‐MLKL and p‐RIPK3. It is speculated that mitochondrial ROS generation can result in necroptosis, but is bypassed, activating the necroptotic pathway downstream at the RIPK3 or MLKL expression level.^41^ In this study, it was identified that ketamine alleviated TNF‐α‐induced necroptosis of hippocampal neurons both in vitro and in vivo, which were indicated to be associated with the inhibition of MLKL phosphorylation and ROS levels. However, the underlying mechanism by which activated MLKL kills cells and the role of ketamine remains unclear, and further studies are required to clarify the process of necroptosis in hippocampal neurons. In conclusion, the results presented in this study provide a clinical context for the inhibitory effect of ketamine on TNF‐α‐induced necroptosis.

Microglia, the resident macrophages of the CNS, are activated by a limited number of stimuli, including lipopolysaccharide, which can mediate neuroinflammation.^42^ Although microglia have long been considered to be crucial players in generating and maintaining inflammatory responses in the CNS, accumulating evidence clearly shows that they have more diverse functions in both the healthy and the injured brain.^43^, ^44^ Innate immune responses and phagocytosis represent a portion of the microglial functional repertoire in terms of the expression of numerous receptors, cell surface molecules, and proteins that enable bidirectional interactions with other cell types in the brain.^28^, ^45^ Iba‐1, which is expressed exclusively in microglia/macrophages,^46^ and the CD68 marker are most commonly used for discerning macrophages immunohistochemically. Using Iba‐1 and CD68 double‐labelling, this study was able to confirm the physiological state of microglia based on morphology and immunoreactivity.^47^ Immunohistochemical analysis of Iba‐1 and CD68 revealed that microglia were activated in the in vivo TNF‐α‐induced SIRS model; moreover, exacerbated neuronal dysfunction was verified and found to be attenuated by ketamine pre‐treatment. Further investigation is required regarding microglial involvement in the regulation of neurons and the role of ketamine in this process.

Overall, this study provided experimental evidence that necroptosis of hippocampal neurons may be induced by TNF‐α both in vivo and in vitro, and could be attenuated by ketamine via inhibition of ROS production and MLKL phosphorylation.

## CONFLICT OF INTEREST

The authors declare that the research was conducted in the absence of any commercial or financial relationships that could be construed as a potential conflict of interest.

## AUTHOR CONTRIBUTION

**Lu Wang:** Data curation (lead); Formal analysis (equal); Investigation (lead); Writing‐original draft (lead); Writing‐review & editing (lead). **Bin Deng:** Conceptualization (equal); Funding acquisition (equal); Project administration (lead); Writing‐review & editing (lead). **Panpan Yan:** Formal analysis (equal); Methodology (equal); Writing‐original draft (equal). **Huanghui Wu:** Methodology (equal); Validation (equal); Writing‐review & editing (supporting). **Chunhui Li:** Data curation (equal); Methodology (equal). **Hongrui Zhu:** Investigation (equal); Methodology (equal). **Jiwei Du:** Conceptualization (lead); Project administration (equal); Validation (equal). **Lichao Hou:** Conceptualization (lead); Funding acquisition (lead); Project administration (equal); Resources (equal); Writing‐review & editing (supporting).

## Supporting information

Fig S1‐S2

## Data Availability

All datasets generated for this study are included in the manuscript.

## References

[1] Davies MGHP‐O . Systemic inflammatory response syndrome. Br J Surg. 1997;84:920‐935. 9240130 10.1002/bjs.1800840707

[2] Cohen J . The immunopathogenesis of sepsis. Nature. 2002;420(6917):885‐891. 12490963 10.1038/nature01326

[3] Singer M , Deutschman CS , Seymour CW , et al. The third international consensus definitions for sepsis and septic shock (Sepsis‐3). JAMA. 2016;315:801‐810. 26903338 10.1001/jama.2016.0287 PMC4968574

[4] Liu V , Escobar GJ , Greene JD , et al. Hospital deaths in patients with sepsis from 2 independent cohortshospital deaths in patients with sepsisletters. JAMA. 2014;312:90‐92. 24838355 10.1001/jama.2014.5804

[5] Iwashyna TJ , Ely EW , Smith DM , Langa KM . Long‐term cognitive impairment and functional disability among survivors of severe sepsis. JAMA. 2010;304:1787‐1794. 20978258 10.1001/jama.2010.1553 PMC3345288

[6] Montgomery SL , Bowers WJ . Tumor necrosis factor‐alpha and the roles it plays in homeostatic and degenerative processes within the central nervous system. J Neuroimmune Pharmacol. 2012;7:42‐59. 21728035 10.1007/s11481-011-9287-2

[7] Liu S , Wang X , Li Y , et al. Necroptosis mediates TNF‐induced toxicity of hippocampal neurons. Biomed Res Int. 2014;2014:290182. 25093162 10.1155/2014/290182 PMC4100394

[8] He S , Wang L , Miao L , et al. Receptor interacting protein kinase‐3 determines cellular necrotic response to TNF‐alpha. Cell. 2009;137:1100‐1111. 19524512 10.1016/j.cell.2009.05.021

[9] Cho YS , Challa S , Moquin D , et al. Phosphorylation‐driven assembly of the RIP1‐RIP3 complex regulates programmed necrosis and virus‐induced inflammation. Cell. 2009;137:1112‐1123. 19524513 10.1016/j.cell.2009.05.037 PMC2727676

[10] Sun L , Wang H , Wang Z , et al. Mixed lineage kinase domain‐like protein mediates necrosis signaling downstream of RIP3 kinase. Cell. 2012;148:213‐227. 22265413 10.1016/j.cell.2011.11.031

[11] Wang H , Sun L , Su L , et al. Mixed lineage kinase domain‐like protein MLKL causes necrotic membrane disruption upon phosphorylation by RIP3. Mol Cell. 2014;54:133‐146. 24703947 10.1016/j.molcel.2014.03.003

[12] Duprez L , Takahashi N , Van Hauwermeiren F , et al. RIP kinase‐dependent necrosis drives lethal systemic inflammatory response syndrome. Immunity. 2011;35:908‐918. 22195746 10.1016/j.immuni.2011.09.020

[13] Vanden Berghe T , Demon D , Bogaert P , et al. Simultaneous targeting of IL‐1 and IL‐18 is required for protection against inflammatory and septic shock. Am J Respir Crit Care Med. 2014;189:282‐291. 24456467 10.1164/rccm.201308-1535OC

[14] Newton K , Dugger DL , Maltzman A , et al. RIPK3 deficiency or catalytically inactive RIPK1 provides greater benefit than MLKL deficiency in mouse models of inflammation and tissue injury. Cell Death Differ. 2016;23:1565‐1576. 27177019 10.1038/cdd.2016.46 PMC5072432

[15] Zanos P , Moaddel R , Morris PJ , et al. Ketamine and ketamine metabolite pharmacology: insights into therapeutic mechanisms. Pharmacol Rev. 2018;70:621‐660. 29945898 10.1124/pr.117.015198 PMC6020109

[16] Murrough JW , Abdallah CG , Mathew SJ . Targeting glutamate signalling in depression: progress and prospects. Nat Rev Drug Discov. 2017;16:472‐486. 28303025 10.1038/nrd.2017.16

[17] Zhang Z , Zhang L , Zhou C , Wu H . Ketamine inhibits LPS‐induced HGMB1 release in vitro and in vivo. Int Immunopharmacol. 2014;23:14‐26. 25133650 10.1016/j.intimp.2014.08.003

[18] Dale O , Somogyi AA , Li Y , Sullivan T , Shavit Y . Does intraoperative ketamine attenuate inflammatory reactivity following surgery? A systematic review and meta‐analysis. Anesth Analg. 2012;115:934‐943. 22826531 10.1213/ANE.0b013e3182662e30

[19] Li N , Lee B , Liu RJ , et al. mTOR‐dependent synapse formation underlies the rapid antidepressant effects of NMDA antagonists. Science. 2010;329:959‐964. 20724638 10.1126/science.1190287 PMC3116441

[20] Kalliolias GD , Ivashkiv LB . TNF biology, pathogenic mechanisms and emerging therapeutic strategies. Nat Rev Rheumatol. 2016;12:49‐62. 26656660 10.1038/nrrheum.2015.169 PMC4809675

[21] Raith EP , Udy AA , Bailey M , et al. Prognostic accuracy of the SOFA Score, SIRS criteria, and qSOFA score for in‐hospital mortality among adults with suspected infection admitted to the intensive care unit. JAMA. 2017;317:290‐300. 28114553 10.1001/jama.2016.20328

[22] Delvaeye T , Wyffels L , Deleye S , et al. Noninvasive whole‐body imaging of phosphatidylethanolamine as a cell death marker using Tc‐Duramycin during TNF‐induced SIRS. J Nucl Med. 2018;59:1140‐1145. 29419481 10.2967/jnumed.117.205815

[23] Yang ZH , Wu XN , He P , et al. A non‐canonical PDK1‐RSK signal diminishes pro‐caspase‐8‐mediated necroptosis blockade. Mol Cell. 2020;80:296‐310.e6. 32979304 10.1016/j.molcel.2020.09.004

[24] Zhang Y , Ding X , Miao C , Chen J . Propofol attenuated TNF‐α‐modulated occludin expression by inhibiting Hif‐1α/ VEGF/ VEGFR‐2/ ERK signaling pathway in hCMEC/D3 cells. BMC anesthesiology. 2019;19:127. 31288745 10.1186/s12871-019-0788-5 PMC6617648

[25] Chen W , Wu J , Li L , et al. Ppm1b negatively regulates necroptosis through dephosphorylating Rip3. Nat Cell Biol. 2015;17:434‐444. 25751141 10.1038/ncb3120 PMC4523090

[26] Pasparakis M , Vandenabeele P . Necroptosis and its role in inflammation. Nature. 2015;517:311‐320. 25592536 10.1038/nature14191

[27] Hildebrand JM , Tanzer MC , Lucet IS , et al. Activation of the pseudokinase MLKL unleashes the four‐helix bundle domain to induce membrane localization and necroptotic cell death. Proc Natl Acad Sci USA. 2014;111:15072‐15077. 25288762 10.1073/pnas.1408987111 PMC4210347

[28] Salter MW , Stevens B . Microglia emerge as central players in brain disease. Nat Med. 2017;23:1018‐1027. 28886007 10.1038/nm.4397

[29] Sun W , Wu X , Gao H , et al. Cytosolic calcium mediates RIP1/RIP3 complex‐dependent necroptosis through JNK activation and mitochondrial ROS production in human colon cancer cells. Free Radic Biol Med. 2017;108:433‐444. 28414098 10.1016/j.freeradbiomed.2017.04.010

[30] Liu T , Sun X , Cao Z . Shikonin‐induced necroptosis in nasopharyngeal carcinoma cells via ROS overproduction and upregulation of RIPK1/RIPK3/MLKL expression. Onco Targets Ther. 2019;12:2605‐2614. 31118661 10.2147/OTT.S200740 PMC6498394

[31] Ardalan M , Rafati AH , Nyengaard JR , Wegener G . Rapid antidepressant effect of ketamine correlates with astroglial plasticity in the hippocampus. Br J Pharmacol. 2017;174:483‐492. 28087979 10.1111/bph.13714 PMC5323512

[32] Cui Y , Yang Y , Dong Y , Hu H . Decoding depression: insights from glial and ketamine regulation of neuronal burst firing in lateral habenula. Cold Spring Harb Symp Quant Biol. 2018;83:141‐150. 30718267 10.1101/sqb.2018.83.036871

[33] Li K , Yang J , Han X . Ketamine attenuates sepsis‐induced acute lung injury via regulation of HMGB1‐RAGE pathways. Int Immunopharmacol. 2016;34:114‐128. 26945830 10.1016/j.intimp.2016.01.021

[34] Yu M , Shao D , Yang R , Feng X , Zhu S , Xu J . Effects of ketamine on pulmonary inflammatory responses and survival in rats exposed to polymicrobial sepsis. J Pharm Pharm Sci. 2007;10:434‐442. 18261365 10.18433/j3rp46

[35] Beilin B , Rusabrov Y , Shapira Y , et al. Low‐dose ketamine affects immune responses in humans during the early postoperative period. Br J Anaesth. 2007;99:522‐527. 17681970 10.1093/bja/aem218

[36] Welters ID , Feurer MK , Preiss V , et al. Continuous S‐(+)‐ketamine administration during elective coronary artery bypass graft surgery attenuates pro‐inflammatory cytokine response during and after cardiopulmonary bypass. Br J Anaesth. 2011;106:172‐179. 21138901 10.1093/bja/aeq341

[37] Bhutta AT , Schmitz ML , Swearingen C , et al. Ketamine as a neuroprotective and anti‐inflammatory agent in children undergoing surgery on cardiopulmonary bypass: a pilot randomized, double‐blind, placebo‐controlled trial. Pediatr Crit Care Med. 2012;13:328‐337. 21926656 10.1097/PCC.0b013e31822f18f9

[38] Kawasaki T , Ogata M , Kawasaki C , Ogata J , Inoue Y , Shigematsu A . Ketamine suppresses proinflammatory cytokine production in human whole blood in vitro. Anesth Analg. 1999;89:665‐669. 10475301 10.1097/00000539-199909000-00024

[39] Getachew B , Aubee JI , Schottenfeld RS , Csoka AB , Thompson KM , Tizabi Y . Ketamine interactions with gut‐microbiota in rats: relevance to its antidepressant and anti‐inflammatory properties. BMC Microbiol. 2018;18:222. 30579332 10.1186/s12866-018-1373-7 PMC6303954

[40] Clarke M , Razmjou S , Prowse N , et al. Ketamine modulates hippocampal neurogenesis and pro‐inflammatory cytokines but not stressor induced neurochemical changes. Neuropharmacology. 2017;112:210‐220. 27106168 10.1016/j.neuropharm.2016.04.021

[41] Moerke C , Bleibaum F , Kunzendorf U , Krautwald S . Combined knockout of RIPK3 and MLKL reveals unexpected outcome in tissue injury and inflammation. Front Cell Dev Biol. 2019;7:19. 30842945 10.3389/fcell.2019.00019 PMC6391322

[42] Ock J , Han HS , Hong SH , et al. Obovatol attenuates microglia‐mediated neuroinflammation by modulating redox regulation. Br J Pharmacol. 2010;159:1646‐1662. 20397299 10.1111/j.1476-5381.2010.00659.x PMC2925488

[43] Norris GT , Kipnis J . Immune cells and CNS physiology: microglia and beyond. J Exp Med. 2019;216:60‐70. 30504438 10.1084/jem.20180199 PMC6314530

[44] Liddelow SA , Guttenplan KA , Clarke LE , et al. Neurotoxic reactive astrocytes are induced by activated microglia. Nature. 2017;541:481‐487. 28099414 10.1038/nature21029 PMC5404890

[45] Li Q , Cheng Z , Zhou L , et al. Developmental heterogeneity of microglia and brain myeloid cells revealed by deep single‐cell RNA sequencing. Neuron. 2019;101:207‐223.e10. 30606613 10.1016/j.neuron.2018.12.006 PMC6336504

[46] Ito D , Imai Y , Ohsawa K , Nakajima K , Fukuuchi Y , Kohsaka S . Microglia‐specific localisation of a novel calcium binding protein, Iba1. Brain Res Mol Brain Res. 1998;57:1‐9. 9630473 10.1016/s0169-328x(98)00040-0

[47] Stankov A , Belakaposka‐Srpanova V , Bitoljanu N , Cakar L , Cakar Z , Rosoklija G . Visualisation of microglia with the use of immunohistochemical double staining method for CD‐68 and Iba‐1 of cerebral tissue samples in cases of brain contusions. Pril (Makedon Akad Nauk Umet Odd Med Nauki). 2015;36:141‐145. 27442380 10.1515/prilozi-2015-0062

