# Supplementary material for: Neuroprotective effect of ketamine against TNF‐α‐induced necroptosis in hippocampal neurons
Source: J Cell Mol Med. 2021 Mar 3;25(7):3449–59. doi: 10.1111/jcmm.16426 (PMC8034479; doi:10.1111/jcmm.16426)
Supplement: Supplementary file 1 — Fig S1‐S2 [file JCMM-25-3449-s001.docx]

Supplemental information

For

Neuroprotective effect of ketamine against TNF-α-induced necroptosis in hippocampal neurons

**Lu Wang^a, 1^, Bin Deng^a, d, 1^, Panpan Yan^b, 1^, Huanghui Wu^a^, Chunhui Li^a^, Hongrui Zhu^a^,** **Jiwei Du^c*^ and Lichao Hou^a*^**.

a Department of Anesthesiology, Xiang'an Hospital of Xiamen University, School of Medicine, Xiamen University. Xiamen 361101, China.

b Medical College of Yan'an University, Yan'an, 716000, China.

c Department of Nursing, Xiang'an Hospital of Xiamen University, School of Medicine, Xiamen University. Xiamen 361101, China.

d State Key Laboratory of Cellular Stress Biology, Xiamen University. Xiamen 361101, China.

*Correspondence:

Jiwei Du, Department of nursing, Xiang’an Hospital, Xiamen University, No. 2000 Xiang’an dong Road, Xiang’an District, Xiamen 361101, China. Email: dujiwei1977@163.com,

Lichao Hou, Department of Anesthesiology, Xiang’an Hospital, Xiamen University, No. 2000 Xiang’an dong Road, Xiang’an District, Xiamen 361101, China. Email: LCHOU@XAH.XMU.EDU.CN

1 Lu Wang, Bin Deng, and Panpan Yan should be considered joint first author.

Supplemental information including:

Figure S1: The non-lethal dose of TNF-α(10μg/per) could cause motor dysfunction in mice.

Figure S2: Ketamine in a dose-dependent manner alleviated motor dysfunction caused by TNF- α-induced SIRS


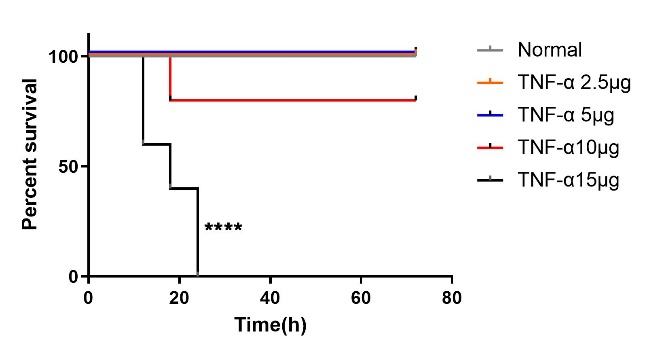

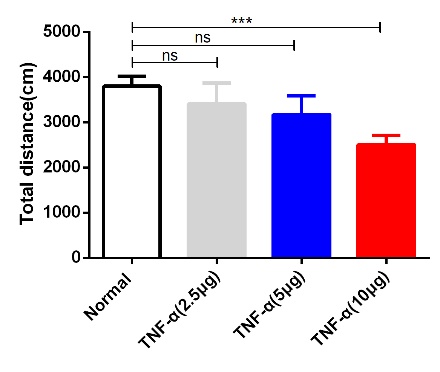


**Figure S1. The non-lethal dose of TNF-α(10μg/per) could cause motor dysfunction in mice.** Survival and the total motion distance in the open field test after 3 days were observed by injecting different concentrations of TNF-α into the tail vein of mice. All data were shown as mean ± s.e.m. n = 7/group, ⁎ significantly different from the normal group; ns, not significant. *** P<0.001.


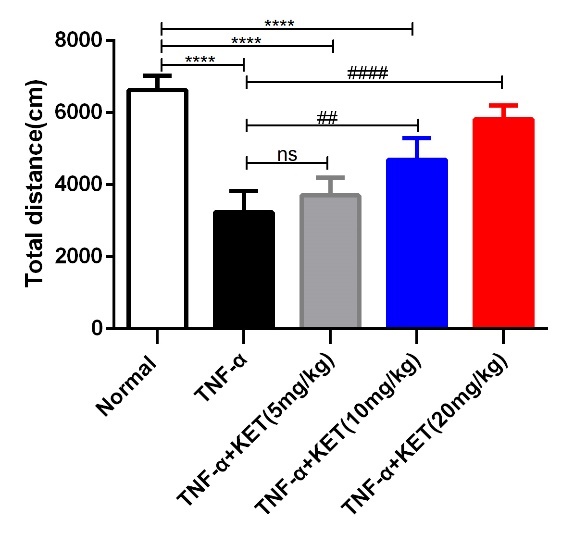

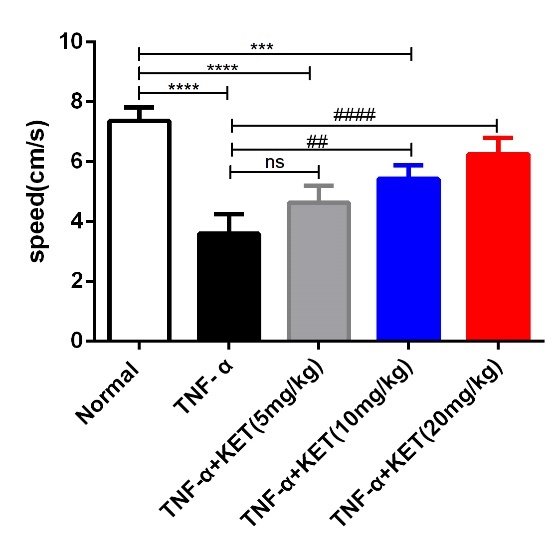


**Figure S2. Ketamine in a dose-dependent manner alleviated motor dysfunction caused by TNF- α-induced SIRS.** The total motion distance and the average motion speed in the open field test after 3 days following tale-vein TNF- α injection and pretreatment with different dose of ketamine. All data were shown as mean ± s.e.m. n = 7/group, ⁎ significantly different from the normal group; # significantly different from the TNF -α group. ****P<0.0001, ##P< 0.01 and ####P< 0.0001.
